# Iron–Manganese-Modified Hydrochar for Synergistic Stabilization of Antimony and Arsenic in Smelter-Impacted Soils

**DOI:** 10.3390/toxics13080674

**Published:** 2025-08-10

**Authors:** Junhuan Wang, Yue Geng, Hong Hou, Xianjun Li

**Affiliations:** 1 State Key Laboratory of Environmental Criteria and Risk Assessment, Chinese Research Academy of Environmental Sciences, Beijing 100012, China; wangjunhuan_1993@163.com (J.W.); 13596634913@163.com (Y.G.); 2 Fujian Key Laboratory of Toxicant and Drug Toxicology, Medical College, Ningde Normal University, Ningde 352100, China

**Keywords:** antimony, arsenic, stabilization

## Abstract

Soil co-contamination with antimony (Sb) and arsenic (As) presents significant ecological and human health risks, demanding effective stabilization solutions. This study evaluated iron–manganese-modified hydrochar (FMHC) for synergistic Sb-As stabilization in contaminated smelter soils. Through 60-day natural aging and 30 accelerated aging cycles, we assessed stabilization performance using toxicity leaching tests (acid/water/TCLP), bioavailable fraction analysis, bioaccessibility assessment, and Wenzel sequential extraction. The key findings reveal that FMHC (5 wt%) achieves durable stabilization: (1) leaching concentrations remained stable post-aging (Sb: 0.3–4.5 mg·L^−1^, >70% stabilization; As: <0.4 mg·L^−1^, >94% stabilization); (2) bioavailable fractions showed maximum reductions of 64% (Sb) and 53% (As), though with some fluctuation; and (3) bioaccessible As was consistently reduced (55–77%), while Sb exhibited greater variability (maximum 58% reduction). Speciation analysis revealed similar stabilization pathways: Sb stabilization resulted from decreased non-specifically and specifically adsorbed fractions, while As stabilization involved the reduction in non-specifically/specifically adsorbed and amorphous to poorly crystalline Fe/Al hydrous oxide-bound fractions. These transformation mechanisms explain FMHC’s superior performance in converting labile Sb/As into stable forms, offering a sustainable solution for the green remediation of Sb-As co-contaminated soils in mining areas.

## 1. Introduction

Antimony (Sb) and arsenic (As), metalloid elements of the VA family, are known to be toxic and carcinogenic. Sb and As co-contamination in soils has become a widespread environmental issue, particularly in regions with intensive mining, smelting, and industrial activities. Both elements often coexist in sulfide ore deposits, leading to their simultaneous release into soils through anthropogenic and geogenic processes. For example, in soils from an abandoned antimony smelting site in Qinglong, Guizhou Province, China, total Sb concentrations reached 27.8–2885 mg·kg^−1^, while As levels were relatively lower at 14.9–63.8 mg·kg^−1^, demonstrating significant potential ecological risks [1]. At another abandoned smelting site in Xihe County, Gansu Province, China, soil Sb concentrations ranged from 148 to 1.85 × 10^5^ mg·kg^−1^, with As levels between 15.7 and 3.69 × 10^3^ mg·kg^−1^. The mean geo-accumulation index (*I*_geo_) values for Sb and As were 10.1 and 1.97, respectively, indicating severe and moderate pollution levels [2]. An epidemiological study revealed that near a typical Sb mining area, 98.82% of local residents exceeded the Sb intake limit, while 63.07% exceeded the As intake threshold. The average daily total intake of Sb and As was 1.08 × 10^−2^ mg·kg^−1^·d^−1^ and 1.19 × 10^−3^ mg·kg^−1^·d^−1^, respectively [3]. In Austria, centuries of ore mining have resulted in elevated concentrations of Sb and As in riverbank soils, reaching up to 49,000 mg·kg^−1^ for As and 2446 mg·kg^−1^ for Sb. Bioaccessibility tests showed maximum values of 317 mg·kg^−1^ for As and 156 mg·kg^−1^ for Sb under gastric conditions, while intestinal conditions yielded up to 298 mg·kg^−1^ for As and 30 mg·kg^−1^ for Sb [4].

Currently, three primary approaches—physical, biological, and chemical methods—are employed for remediating As- and Sb-contaminated soils. Among these, chemical stabilization has garnered significant attention due to its high treatment efficiency, minimal time requirements, and operational simplicity. This process converts As and Sb into stable forms through ion exchange, complexation, precipitation, and sorption onto stabilizing agents [5]. Iron–manganese (Fe-Mn) composites demonstrate considerable potential for stabilizing As and Sb in soils [6]. Mn-based materials exhibit strong oxidative capacity, transforming As and Sb into less toxic pentavalent species (As(V)/Sb(V)); however, their performance is highly dependent on environmental parameters, and amorphous manganese oxide may unexpectedly elevate water-soluble fractions of Sb and As in acidic soils (pH < 7) [7]. In contrast, Fe-based materials display exceptional adaptability to complex soil matrices, providing abundant reactive sites for As/Sb stabilization via mechanisms such as inner-sphere complexation and surface precipitation [8]. Fe-Mn composites provide an effective solution for stabilizing As and Sb in the soil by synergistically combining the advantages of both iron and manganese components. Nevertheless, the practical application of Fe-Mn composites is hindered by inadequate durability and particle agglomeration, which substantially reduce contaminant removal efficiency [9].

To address these limitations, Fe-Mn-modified biochar (FMBC) offers a promising solution by enhancing particle dispersion. Typically, Fe and Mn are co-loaded onto biochar for modification; the porous carbon matrix serves as a carrier for Fe-Mn oxides, where Fe provides adsorption sites while Mn facilitates oxidation. For instance, FMBC serves as a cost-effective yet highly efficient adsorbent for aqueous arsenic removal, where the synergistic action between Mn(III)-mediated oxidation and oxygen-containing functional groups facilitates the conversion of As(III) to As(V), followed by effective immobilization through chemisorption processes [10]. The ferromanganese oxide-impregnated biochar composites mediate the oxidation of As(III) to As(V) and facilitate the formation of Fe-Mn plaques on rice root surfaces, thereby enhancing arsenic adsorption and reducing its accumulation in rice roots [11]. In paddy soils, FMBC elevated the soil redox potential and decreased available As, transforming labile to stable As fractions [12]. The application of FMBC to As-contaminated soils mediated Mn-driven oxidation of As(III) to As(V), enhancing Fe-As complexation and reducing TCLP-leachable As from 3.95 to 0.77 mg·L^−1^ (80.5% decrease) [13]. The 3% (*w*/*w*) MnFe_2_O_4_-modified biochar immobilized 43.5% of extractable Sb in soil by converting exchangeable fractions into non-labile forms [14]. A comparative study demonstrated that FMBC outperformed Fe-Mn-modified activated carbon in stabilizing soil Sb and As, significantly increasing their residual fraction contents by 2–3-fold [15]. Although previous studies have demonstrated the effectiveness of FMBC in stabilizing Sb/As in soils, the evaluation of its stabilization performance has primarily relied on a single criterion, such as short-term leaching toxicity and speciation transformation. Multidimensional comprehensive assessments (e.g., integrating leaching toxicity, bioavailability, bioaccessibility, and long-term stability) remain lacking.

This study systematically evaluated the stabilization performance of Fe-Mn-modified hydrochar for Sb and As in co-contaminated soils through (1) 60-day natural aging and 30-cycle accelerated aging experiments, (2) a multidimensional assessment (leaching toxicity, bioavailability, and bioaccessibility), and (3) speciation analysis to elucidate stabilization mechanisms. The results provide both theoretical guidance and empirical data for field applications of this remediation technology.

## 2. Materials and Methods

### 2.1. Chemicals and Reagents

All chemical reagents used in this study were obtained from commercial suppliers. Specifically, Fe(NO_3_)_3_, KMnO_4_, AsNa_3_O_4_, HCl, NaOH, KOH, NaHCO_3_, HNO_3_, H_2_SO_4_, CH_3_COOH, HF, and HClO_4_ were sourced from Sinopharm Chemical Reagent Co., Ltd. (Shanghai, China). KSb(OH)_6_, Na_2_HPO_4_, pepsin, sodium malate, sodium citrate, lactic acid, bile salts, trypsin, (NH_4_)_2_SO_4_, NH_4_H_2_PO_4_, NH_4_-oxalate, ascorbic acid, thiourea, and KBH_4_ were supplied by Macklin Reagent (Shanghai, China). Certified reference materials (CRMs) for antimony CAS 7440-36-0) and arsenic (CAS 7440-38-2) were acquired from Beijing Northern Weiye Metrology Technology Co., Ltd. (Beijing, China).

### 2.2. Preparation and Modification of Hydrochar

Waste poplar bark was collected from agricultural land in Shandong Province, China. The raw material was air-dried, mechanically crushed, and sieved through a 100-mesh screen. Subsequently, 34 g of bark powder was homogenized with 340 mL of distilled water (1:10 *w*/*v* ratio) in a laboratory-scale reactor (JINGLILONG GR-500/SC-L, Beijing, China). Hydrothermal carbonization was conducted at 230 °C for 4 h under autogenous pressure. The resulting product was repeatedly washed with deionized water to remove ash impurities and then oven-dried at 60 °C to a constant weight to obtain the final hydrochar (HC) product.

To prepare the Fe-Mn-modified hydrochar (FMHC), 2 g of HC was first dispersed in 200 mL of 0.15 M Fe(NO_3_)_3_ solution and mixed thoroughly. Then, 200 mL of 0.05 M KMnO_4_ solution was slowly added under continuous stirring. The resulting mixture was vigorously stirred for 30 min, followed by 2 h of ultrasonication, and subsequently shaken in a water bath at room temperature (25 ± 1 °C) for 12 h. The suspension was then centrifuged at 4000× *g* for 5 min, and the obtained precipitate was repeatedly washed with deionized water until the pH of the supernatant became stable. Finally, the purified product was freeze-dried to a constant weight to obtain the FMHC material.

The preparation methods for the above-mentioned HC and FMHC were based on our unpublished master’s thesis. The optimal hydrothermal carbonization parameters (230 °C for 4 h) were determined through preliminary adsorption experiments to maximize Sb(V)/As(V) removal efficiency, while the molar ratio of Fe/Mn (3:1) was adopted from established studies [16,17].

To facilitate readers’ comprehension, we briefly introduce the basic characteristics of FMHC: Fe and Mn were uniformly loaded on the surface of FMHC in the form of FeOOH, Fe_2_O_3_, and MnO_2_. Fe and Mn doping greatly improved the specific surface area of the hydrochar, significantly increasing from 2.37 to 178.50 m^2^·g^−1^, a 75-fold increase. The maximum adsorption capacity was increased to 81.53 mg·g^−1^ and 153.37 mg·g^−1^ for Sb(V) and As(V), respectively.

### 2.3. Soil Samples and Their Physicochemical Properties

Two soil samples (Soil#1 and Soil#2) were collected from an antimony smelter located in Gansu, China (105°18′ N, 34°02′ E). Table 1 presents the basic physicochemical properties of these soil samples. The collected soil was air-dried and sieved with a 100-mesh sieve. Soil pH was measured with a solid/liquid ratio of 1.0 g:5.0 mL. Electric conductivity (EC) was determined in solutions (filtrated with 0.45 μm nylon membrane) with a solid/liquid ratio of 0.5 g:2.5 mL using a conductivity detector (DDB-12L, Qiwei, Hangzhou, China). Total organic carbon (TOC) was measured through the combustion oxidation-titration method (Environmental protection standards of the People’s Republic of China, HJ 658-2013) [18]. The cation exchange capacity (CEC) was determined via the hexamminecobalt trichloride solution spectrophotometric method (Environmental protection standards of the People’s Republic of China, HJ 889-2017) [19]. The distribution of soil particle size was investigated via a laser particle size analyzer (HELOS-RODOS, SYMPATEC GmbH, Clausthal-Zellerfeld, Germany). The total content of Sb/As in the soils was digested with HF–HNO_3_–HClO_4_ (*v*/*v*/*v* = 1:3:2).

### 2.4. Extraction and Detection of Antimony and Arsenic in Soil

A comprehensive assessment of antimony and arsenic in soil was conducted, encompassing leaching toxicity, bioavailability, bioaccessibility, and speciation analysis (detailed in Table 2). After extraction, leachates were filtered through 0.45 μm nylon syringe filters to remove suspended particulates. Prior to analysis, the filtered extracts underwent a two-step pretreatment protocol: (1) dilution with 5% (*v*/*v*) HCl solution to achieve optimal analyte concentrations within the instrument detection range, followed by (2) chemical reduction using 10 g·L^−1^ thiourea at 60 °C for 30 min to convert Sb(V) and As(V) species to their trivalent forms. The total Sb and As concentrations were quantified via hydride generation atomic fluorescence spectrometry (HG-AFS, AFS-933, Titan Instruments, Beijing, China) with optimized excitation wavelengths of 217.6 nm (Sb) and 193.7 nm (As). Standard curves were drawn using CRMs of Sb and As. Quality assurance measures included procedural blanks and triplicate sampling with relative standard deviations < 5%.

### 2.5. Stabilization of Sb and As in Soil of Antimony Mining Area: Short-Term and Long-Term Effects

#### 2.5.1. Effect of Material Dosage on Short-Term Stabilization Effect

The same amount of HC and FMHC (1 g) was applied to various amounts of contaminated soils (50 g, 40 g, 30 g, 20 g, and 10 g). Different dosages of the stabilizing agent were supplemented: 2%, 2.5%, 3.3%, 5%, and 10%. Subsequently, the contaminated soils and the amended hydrochars were mixed thoroughly in a 25 mL plastic container with a lid. The mixture was then kept at 70% field water capacity using distilled water for 40 days at room temperature, while stirring was conducted every three days during the passivation period. The control soils lacked a stabilizer. All treatments were performed in triplicate. The stabilization efficiency was determined by the reduction in the water leachable Sb/As compared to control soils.

#### 2.5.2. Comprehensive Evaluation of the Short-Term Stabilization Effect of Sb/As by FMHC

Soil samples (60 g) were amended with 3 g of FMHC and 30 mL deionized water to achieve 50% moisture content (*w*/*w*). During the 60-day incubation period, daily additions of deionized water were made to maintain constant moisture levels through gravimetric adjustment. Subsamples were collected at 7, 15, 30, and 60 days post-treatment for subsequent analysis. Parallel control groups without FMHC amendment were established under identical experimental conditions. All treatments were conducted in triplicate.

#### 2.5.3. Comprehensive Evaluation of the Long-Term Stabilization Effect of Sb/As by FMHC

Furthermore, the long-term stabilization effect of Sb/As by FMHC was assessed through laboratory-accelerated aging tests, involving 30 dry–wet cycles (16 h wetting followed by 8 h oven drying at 40 °C per cycle) and 30 freeze–thaw cycles (16 h at 30 °C and 8 h at −20 °C per cycle). All treatments were conducted in triplicate.

### 2.6. Figures

All figures were generated using GraphPad Prism 9 and Origin 2024. Analysis of variance (ANOVA) and correlation (Pearson) were carried out with IBM SPSS statistics 20 software. Differences between groups/treatments with a *p*-value < 0.05 (LSD) were considered statistically significant.

## 3. Results

### 3.1. Stabilization of Sb/As in Soil by HC and FMHC: Effect of Dosage

**Figure 1:**
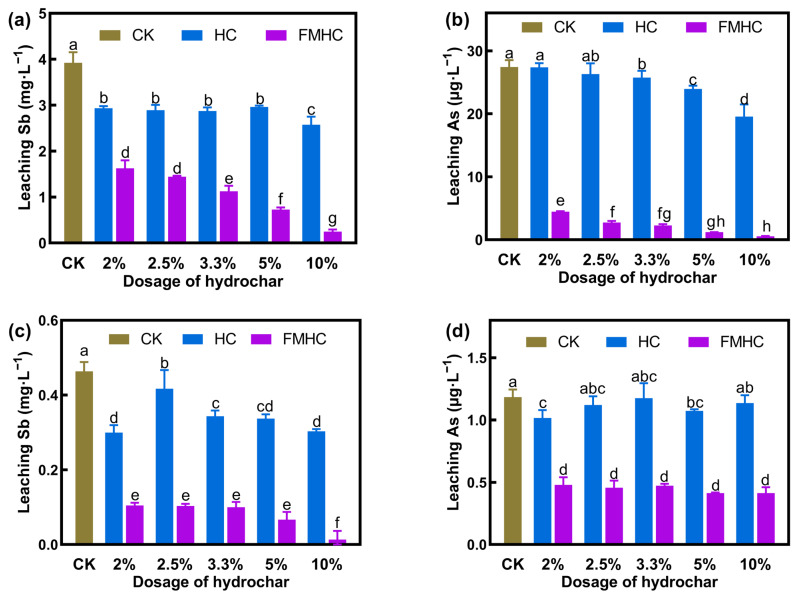
Stabilization of Sb and As in soil by HC and FMHC. Water leaching Sb (**a**) and As (**b**) in Soil#1. Water leaching Sb (**c**) and As (**d**) in Soil#2. CK represents the soils without amendment. Different lowercase letters indicate significant differences between groups (*p* < 0.05).

The short-term stabilization efficacy of HC and FMHC was evaluated by the reduction in leachable Sb/As. To clarify, distilled water was used to extract the leachable Sb/As to stimulate natural leaching conditions. Two soil samples contaminated with different levels of antimony and arsenic were utilized. Soil#1 contained a total of 661.98 and 51.79 mg·kg^−1^ Sb and As, respectively, whereas Soil#2 contained 238.69 and 25.04 mg·kg^−1^ Sb and As, respectively. Different dosages of HC and FMHC were amended in Soil#1 and Soil#2, and the leached Sb and As were measured after 40 days of stabilization. Figure 1 displays the results.

The concentration of leached As in untreated soil was low, 27.44 μg·L^−1^ in Soil#1 and 1.18 μg·L^−1^ in Soil#2. The amendment of HC showed little effect on the stabilization of As. With HC applied at dosages of 2%~10%, the leached As remained stable, ranging from 19.55 to 27.36 μg·L^−1^ in Soil#1 and 1.01 to 1.14 μg·L^−1^ in Soil#2. The highest As stabilization efficiency (28.75%) was achieved with the addition of 10% HC in Soil#1. Conversely, the application of FMHC significantly reduced the leachability of As. Passivated with FMHC at dosages of 2%~10%, the leached As was notably reduced, varying from 0.55 to 4.47 μg·L^−1^ in Soil#1 and 0.41 to 0.48 μg·L^−1^ in Soil#2. The stabilization efficiency of As increased with the addition of FMHC in Soil#1. While in Soil#2, the stabilization efficiency of As was about 60%, regardless of the dose. The supplement of 2% FMHC could stabilize 83.71% of As in Soil#1, whereas 10% FMHC was able to alleviate 97.99% of As. Although the pristine HC failed to stabilize As in soil, the modified FMHC could sufficiently alleviate As.

The concentrations of leached Sb in Soil#1 and Soil#2 were 3.92 and 0.46 mg·L^−1^, respectively. HC exhibited limited ability to stabilize Sb. There was no dose–effect relationship between HC addition and Sb stabilization efficiency. In Soil#1, the stabilization efficiency of Sb was stable at about 30% (24.46%~34.46%), regardless of how much HC was added. Similarly, the lowest Sb stabilization efficiency (8.84%) occurred when 2.5% of HC was applied in Soil#2. When supplemented with 2%, 3.3%, 5.0%, and 10.0% HC, 25.74%~35.17% of Sb was alleviated. FMHC exhibited significantly better remediation performance, with doses of 2%~10%, significantly reducing the leached Sb, varying from 0.25 to 1.63 mg·L^−1^ in Soil#1 and 0.01 to 0.11 mg·L^−1^ in Soil#2. Evidently, the stabilization efficiency of Sb increased with the dosage of FMHC in both test soils. The maximum passivation efficiency of Sb was achieved with the application of 10% FMHC, 93.66% in Soil#1 and 96.99% in Soil#2. While the pristine HC performed poorly in passivating Sb and As, the modified FMHC could effectively immobilize Sb and As in the co-contaminated soil.

### 3.2. Stabilization Effect of Sb/As by FMHC: Short-Term and Long-Term

To comprehensively assess the stabilization performance of FMHC on antimony and arsenic in the soil, FMHC at a mass ratio of 5% was added to the soil, and the stabilization effect was evaluated from multiple aspects, including leaching toxicity (water, HNO_3_-H_2_SO_4,_ and TCLP), bioavailability (Na_2_HPO_4_), and bioaccessibility (PBET). The short-term stabilization effect was evaluated by the changes in antimony and arsenic after 60 days of natural aging, and the long-term stabilization potential was evaluated through accelerated aging experiments (30 cycles of dry–wet and freeze–thaw).

#### 3.2.1. Evaluation Based on Leaching Toxicity

**Figure 2:**
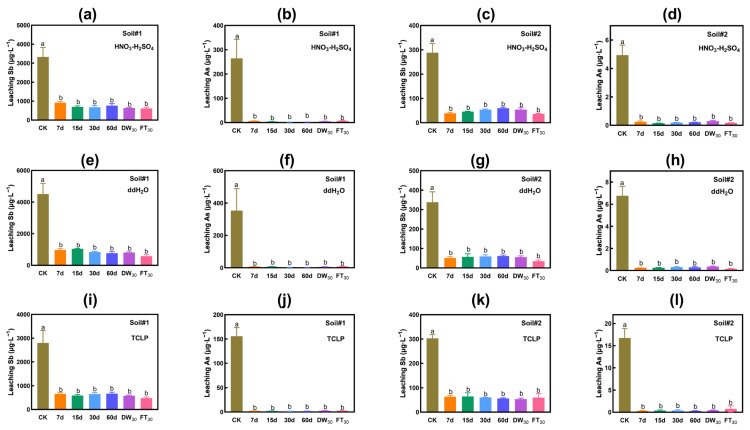
Leaching toxicity of antimony and arsenic in soil. HNO_3_-H_2_SO_4_ leaching Sb (**a**) and As (**b**) in Soil#1. HNO_3_-H_2_SO_4_ leaching Sb (**c**) and As (**d**) in Soil#2. Water leaching Sb (**e**) and As (**f**) in Soil#1. Water leaching Sb (**g**) and As (**h**) in Soil#2. TCLP leaching Sb (**i**) and As (**j**) in Soil#1. TCLP leaching Sb (**k**) and As (**l**) in Soil#2. CK represents untreated control soil samples without amendment. Samples labeled 7d, 15d, 30d, and 60d denote specimens amended with 5% FMHC and cured for 7, 15, 30, and 60 days, respectively, under natural conditions. DW_30_ and FT_30_ indicate modified soil samples subjected to environmental stress testing through 30 cycles of alternate wet–dry conditions and freeze–thaw treatments, respectively. Different lowercase letters indicate significant differences between groups (*p* < 0.05).

Figure 2a–d displays the changes in leaching concentration extracted by HNO_3_-H_2_SO_4_. The amendment of FMHC significantly decreased the acid leaching concentrations of antimony and arsenic in the tested soils and maintained a stable effect during 60 days of short-term natural aging and 30 rounds of accelerated aging. In Soil#1, the leaching concentration of antimony dropped from 3326 μg·L^−1^ to 615–916 μg·L^−1^, and the stability rate was 72–82%. The leaching concentration of arsenic decreased from 264 μg·L^−1^ to 1.9–6.8 μg·L^−1^, with a stability rate higher than 97%. The leaching concentration of arsenic after remediation met the limit for drinking water (<10 μg·L^−1^, World Health Organization, Guidelines for drinking-water quality). In Soil#2, the leaching concentration of antimony declined from 288 μg·L^−1^ to 36–60 μg·L^−1^, and the stability rate was 79–87%. The leaching concentration of arsenic decreased from 4.9 μg·L^−1^ to 0.1–0.3 μg·L^−1^, with a stability rate higher than 94%. The leaching concentrations of arsenic both before and after treatment met the requirements for domestic drinking water.

The result of water leaching is in line with that of acid leaching (Figure 2e–h). In Soil#1, the leaching concentration of antimony dropped from 4514 μg·L^−1^ to 582–1044 μg·L^−1^, and the stability rate was 77–87%. The leaching concentration of arsenic declined from 353 μg·L^−1^ to 1.9–8.8 μg·L^−1^, with a stability rate higher than 97%. The leaching concentration of arsenic after remediation met the limit requirements for drinking water. In Soil#2, the leaching concentration of antimony decreased from 338 μg·L^−1^ to 35–60 μg·L^−1^, and the stability rate was 82–90%. The leaching concentration of arsenic dropped from 6.8 μg·L^−1^ to 0.2–0.4 μg·L^−1^, with a stability rate higher than 95%. The leaching concentrations of arsenic both before and after treatment met the requirements for domestic drinking water.

Figure 2i–l depicts the variation in the leaching concentration of antimony and arsenic extracted by the TCLP method. In line with water extraction/HNO_3_-H_2_SO_4_ extraction, the addition of FMHC significantly mitigated the leaching toxicity of antimony and arsenic in the soil and maintained stable outcomes under 60 days of short-term natural aging and 30 rounds of accelerated aging. In Soil#1, the leaching concentration of antimony dropped from 2799 μg·L^−1^ to 481–671 μg·L^−1^, and the stability rate was 76–83%. The leaching concentration of arsenic decreased from 156 μg·L^−1^ to 1.3–2.4 μg·L^−1^, with a stability rate exceeding 98%. The leaching concentration of arsenic after remediation met the limit requirements for drinking water. In Soil#2, the leaching concentration of antimony declined from 303 μg·L^−1^ to 54–64 μg·L^−1^, and the stability rate was 79–82%. The leaching concentration of arsenic decreased from 16.7 μg·L^−1^ to 0.3–0.8 μg·L^−1^, with a stability rate greater than 95%. The leaching concentration of arsenic after treatment fulfilled the requirements for domestic drinking water.

#### 3.2.2. Evaluation Based on Bioavailability

**Figure 3:**
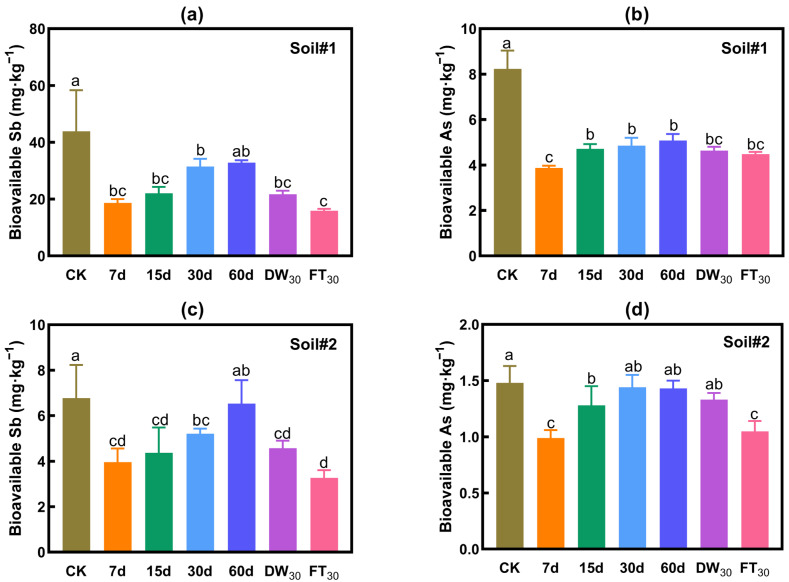
Bioavailability of antimony and arsenic in soil: Na_2_HPO_4_ extraction. Bioavailable Sb (**a**) and As (**b**) in Soil#1. Bioavailable Sb (**c**) and As (**d**) in Soil#2. CK represents untreated control soil samples without amendment. Samples labeled 7d, 15d, 30d, and 60d denote specimens amended with 5% FMHC and cured for 7, 15, 30, and 60 days, respectively, under natural conditions. DW_30_ and FT_30_ indicate modified soil samples subjected to environmental stress testing through 30 cycles of alternate wet–dry conditions and freeze–thaw treatments, respectively. Different lowercase letters indicate significant differences between groups (*p* < 0.05).

Figure 3 presents the variations in bioavailable concentrations of antimony and arsenic in soil. The addition of FMHC significantly reduces the bioavailable concentrations of these metalloids, achieving maximum reduction rates of 64% for Sb and 53% for As. During the aging process, the bioavailable Sb/As concentrations exhibit fluctuations that may compromise stabilization effectiveness. Under natural aging conditions, bioavailable concentrations of both metalloids show a gradual increase over time, leading to progressively diminished stabilization effects after 30 and 60 days. Comparative analysis reveals that accelerated aging generally results in lower bioavailable concentrations than 60-day natural aging. Notably, freeze–thaw cycling demonstrates superior effectiveness in reducing bioavailable concentrations compared to wet–dry cycling for both Sb and As.

Soil#1: The initial bioavailable Sb concentration was 43.9 mg·kg^−1^. After 7 days of natural aging with FMHC, it decreased to 18.7 mg·kg^−1^, corresponding to a stabilization rate of 58%. However, as natural aging progressed, the bioavailable Sb concentration gradually rose—reaching 31.5 mg·kg^−1^ at 30 days (28% stabilization) and 32.8 mg·kg^−1^ at 60 days (no significant difference from the control, based on triplicate measurements). Accelerated aging, however, reduced the bioavailable Sb concentration to 15.9–21.7 mg·kg^−1^ (50–64% stabilization), with the lowest value observed after 30 freeze–thaw cycles.

Soil#2: The initial bioavailable Sb concentration was 6.8 mg·kg^−1^. Following FMHC addition and 7 days of natural aging, it decreased to 4.0 mg·kg^−1^ (41% stabilization). With prolonged aging, the concentration increased to 5.2 mg·kg^−1^ at 30 days (23% stabilization) and 6.5 mg·kg^−1^ at 60 days (near-zero stabilization effect). Accelerated aging lowered the bioavailable Sb concentration to 3.3–4.6 mg·kg^−1^ (32–52% stabilization), with the minimum occurring after 30 freeze–thaw cycles.

Soil#1: The initial bioavailable As concentration was 8.2 mg·kg^−1^. After 7 days of natural aging with FMHC, it declined to 3.9 mg·kg^−1^ (53% stabilization). Although the concentration increased slightly with extended aging, a reasonable stabilization effect persisted—reaching 5.1 mg·kg^−1^ (38% stabilization) at 60 days. Accelerated aging further reduced the concentration to 4.5–4.6 mg·kg^−1^ (44–46% stabilization). The lowest bioavailable As concentration was recorded after 7 days of natural aging.

Soil#2: The initial bioavailable As concentration was 1.5 mg·kg^−1^. After 7 days of natural aging with FMHC, it decreased to 1.0 mg·kg^−1^ (33% stabilization). However, as aging continued, the concentration rose to 1.3 mg·kg^−1^ at 15 days (14% stabilization) and 1.4 mg·kg^−1^ at 30/60 days (no significant difference from the control). Dry–wet cycling had negligible effects (1.3 mg·kg^−1^), whereas freeze–thaw cycles reduced the concentration to 1.0 mg·kg^−1^ (29% stabilization). The lowest bioavailable As concentration occurred after 7 days of natural aging and 30 freeze–thaw cycles.

#### 3.2.3. Evaluation Based on Bioaccessibility

**Figure 4:**
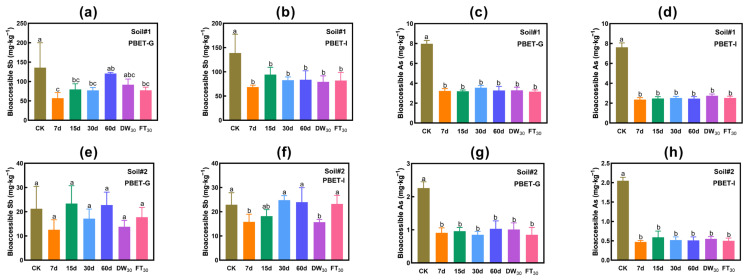
Bioaccessibility of antimony and arsenic in soil: PBET extraction. Gastric phase (PBET-G) extracted Sb (**a**) and As (**c**) in Soil#1, and Sb (**e**) and As (**g**) in Soil#2. Intestinal phase (PBET-I) extracted Sb (**b**) and As (**d**) in Soil#1, and Sb (**f**) and As (**h**) in Soil#2. CK represents untreated control soil samples without amendment. Samples labeled 7d, 15d, 30d, and 60d denote specimens amended with 5% FMHC and cured for 7, 15, 30, and 60 days, respectively, under natural conditions. DW_30_ and FT_30_ indicate modified soil samples subjected to environmental stress testing through 30 cycles of alternate wet–dry conditions and freeze–thaw treatments, respectively. Different lowercase letters indicate significant differences between groups (*p* < 0.05).

Figure 4 presents the concentration variations in bioaccessible antimony and arsenic in soil. The addition of FMHC demonstrates effective and sustainable stabilization of arsenic, whereas its stabilizing effect on antimony proves inconsistent. During the aging process, the bioaccessible Sb concentration exhibits irregular fluctuations, leading to reduced stabilization efficacy.

In Soil#1, the initial PBET gastric phase Sb concentration measured 135.7 mg·kg^−1^. Following FMHC application and natural aging (7–30 days), gastric phase Sb concentrations declined to 56.1–79.6 mg·kg^−1^, representing stabilization rates of 41–58%. However, after 60 days of natural aging, concentrations rebounded to 120.1 mg·kg^−1^, showing no significant difference from the FMHC-free control group. Similarly, 30 cycles of wet–dry alternation produced no significant reduction compared to controls. Notably, freeze–thaw cycling reduced gastric phase Sb to 77.4 mg·kg^−1^, achieving a 43% stabilization rate. In contrast, FMHC consistently lowered intestinal phase Sb concentrations from 138.8 mg·kg^−1^ to 68.5–94.3 mg·kg^−1^, maintaining stabilization rates of 32–51% throughout all aging conditions.

In Soil#2, the initial PBET gastric phase Sb concentration (21.2 mg·kg^−1^) showed no significant reduction post-FMHC treatment, fluctuating between 12.6 and 23.4 mg·kg^−1^. For intestinal phase Sb (initial 22.9 mg·kg^−1^), concentrations decreased to 15.8 mg·kg^−1^ (31% stabilization) after 7-day aging but gradually increased to 18.2–24.0 mg·kg^−1^ with extended aging (15–60 days), ultimately showing no significant difference from controls. Dry–wet cycling reduced concentrations to 15.7 mg·kg^−1^ (31% stabilization), while freeze–thaw cycling restored values to control levels (23.2 mg·kg^−1^).

FMHC demonstrated highly consistent arsenic stabilization in the tested soils. In Soil#1, gastric phase As concentrations were reduced from 8.0 mg·kg^−1^ to 3.2–3.6 mg·kg^−1^ (56–60% stabilization), while intestinal phase As decreased from 7.6 mg·kg^−1^ to 2.4–2.7 mg·kg^−1^ (64–69% stabilization). Similarly, in Soil#2, gastric phase As concentrations declined from 2.3 mg·kg^−1^ to 0.8–1.0 mg·kg^−1^ (55–62% stabilization), with intestinal phase As showing a reduction from 2.0 mg·kg^−1^ to 0.5–0.6 mg·kg^−1^ (71–77% stabilization).

### 3.3. Stabilization Mechanism of Sb/As via FMHC: Change in Formation

The stabilization mechanism of FMHC for antimony and arsenic in soil was investigated through speciation analysis. FMHC treatment primarily reduced the labile fractions of these metalloids, with antimony showing decreases in F1 (non-specifically sorbed) and F2 (specifically sorbed) forms, while arsenic exhibited reductions across F1, F2, and F3 (amorphous to poorly crystalline Fe/Al hydrous oxide-bound) fractions.

**Figure 5:**
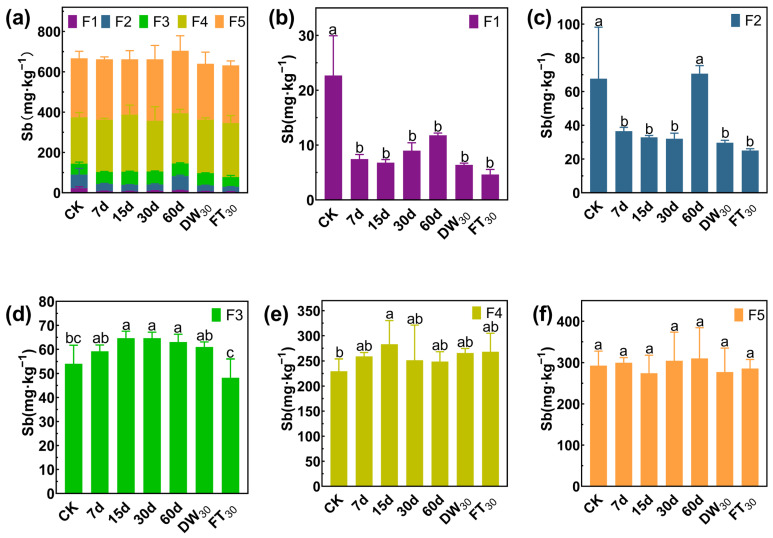
Formation of antimony in Soil#1: Wenzel extraction. (**a**) Column chart of Sb speciation distribution in soil; Concentration changes of F1 (**b**), F2 (**c**), F3 (**d**), F4 (**e**) and F5 (**f**) fraction Sb. CK represents untreated control soil samples without amendment. Samples labeled 7d, 15d, 30d, and 60d denote specimens amended with 5% FMHC and cured for 7, 15, 30, and 60 days, respectively, under natural conditions. DW_30_ and FT_30_ indicate modified soil samples subjected to environmental stress testing through 30 cycles of alternate wet–dry conditions and freeze–thaw treatments, respectively. The five chemical fractions of antimony are operationally defined as follows: F1: non-specifically sorbed (easily mobilizable, outer-sphere complexes); F2: specifically sorbed (readily mobilizable, inner-sphere complexes); F3: amorphous to poorly crystalline Fe/Al hydrous oxide-bound fraction; F4: well-crystallized Fe/Al hydrous oxide-bound fraction; and F5: residual fraction. Different lowercase letters indicate significant differences between groups (*p* < 0.05).

**Figure 6:**
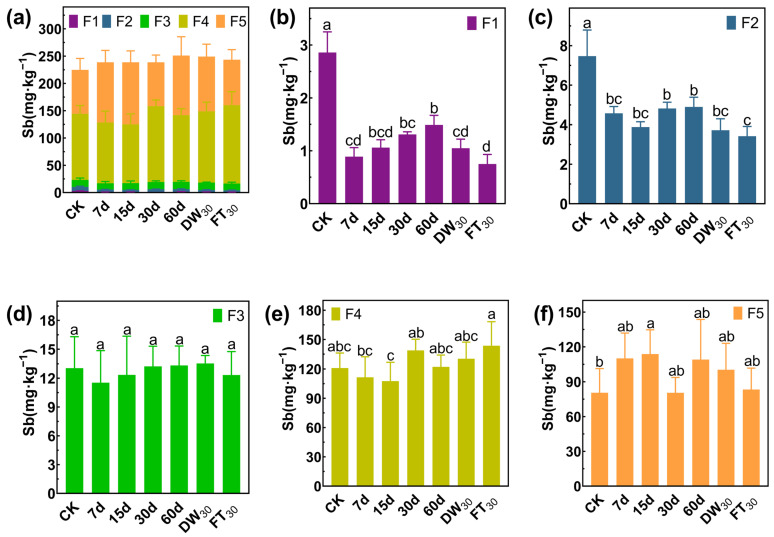
Formation of antimony in Soil#2: Wenzel extraction. (**a**) Column chart of Sb speciation distribution in soil; Concentration changes of F1 (**b**), F2 (**c**), F3 (**d**), F4 (**e**) and F5 (**f**) fraction Sb. CK represents untreated control soil samples without amendment. Samples labeled 7d, 15d, 30d, and 60d denote specimens amended with 5% FMHC and cured for 7, 15, 30, and 60 days, respectively, under natural conditions. DW_30_ and FT_30_ indicate modified soil samples subjected to environmental stress testing through 30 cycles of alternate wet–dry conditions and freeze–thaw treatments, respectively. The five chemical fractions of antimony are operationally defined as follows: F1: non-specifically sorbed (easily mobilizable, outer-sphere complexes); F2: specifically sorbed (readily mobilizable, inner-sphere complexes); F3: amorphous to poorly crystalline Fe/Al hydrous oxide-bound fraction; F4: well-crystallized Fe/Al hydrous oxide-bound fraction; and F5: residual fraction. Different lowercase letters indicate significant differences between groups (*p* < 0.05).

In Soil#1, antimony mainly exists in the forms of F4 and F5, accounting for 78.62%, while the proportions of F1, F2, and F3 are 3.36%, 9.92% and 8.10%, respectively (Figure 5). Amended with FMHC, the F1 fraction decreased from 22.7 mg·kg^−1^ to 4.6–11.8 mg·kg^−1^ (48–80% reduction), while the F2 fraction declined from 67.7 mg·kg^−1^ to 32.0–36.6 mg·kg^−1^ (46–53% reduction) during 7–30 days of aging. However, after 60 days of natural aging, the F2 concentration rebounded to 70.6 mg·kg^−1^, comparable to untreated controls. Subsequent accelerated aging reduced F2 levels to 25.1–29.7 mg·kg^−1^ (56–63% reduction). In Soil#2 (Figure 6), antimony mainly exists in the forms of F4 and F5, accounting for 89.57%, while the proportions of F1, F2, and F3 are 1.28%, 3.33% and 5.83%, respectively. Amended with FMHC, similar patterns were observed with F1 decreasing from 2.9 mg·kg^−1^ to 0.8–1.5 mg·kg^−1^ (48–74% reduction) and F2 from 7.5 mg·kg^−1^ to 3.4–4.9 mg·kg^−1^ (34–54% reduction). The transformation patterns indicated conversion of labile antimony fractions into more stable F4 (well-crystallized Fe/Al hydrous oxide-bound) and F5 (residual) forms, though the high background concentrations of these stable fractions masked detectable increases.

**Figure 7:**
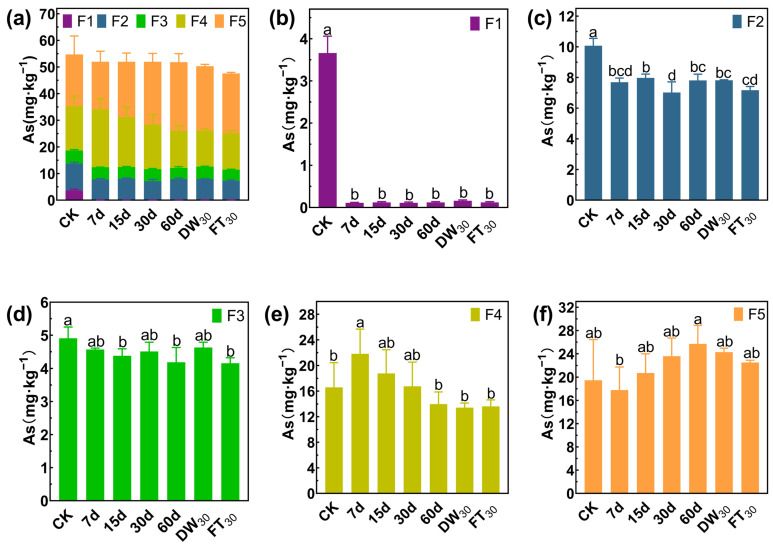
Formation of arsenic in Soil#1: Wenzel extraction. (**a**) Column chart of As speciation distribution in soil; Concentration changes of F1 (**b**), F2 (**c**), F3 (**d**), F4 (**e**) and F5 (**f**) fraction As. CK represents untreated control soil samples without amendment. Samples labeled 7d, 15d, 30d, and 60d denote specimens amended with 5% FMHC and cured for 7, 15, 30, and 60 days, respectively, under natural conditions. DW_30_ and FT_30_ indicate modified soil samples subjected to environmental stress testing through 30 cycles of alternate wet–dry conditions and freeze–thaw treatments, respectively. The five chemical fractions of arsenic are operationally defined as follows: F1: non-specifically sorbed (easily mobilizable, outer-sphere complexes); F2: specifically sorbed (readily mobilizable, inner-sphere complexes); F3: amorphous to poorly crystalline Fe/Al hydrous oxide-bound fraction; F4: well-crystallized Fe/Al hydrous oxide-bound fraction; and F5: residual fraction. Different lowercase letters indicate significant differences between groups (*p* < 0.05).

**Figure 8:**
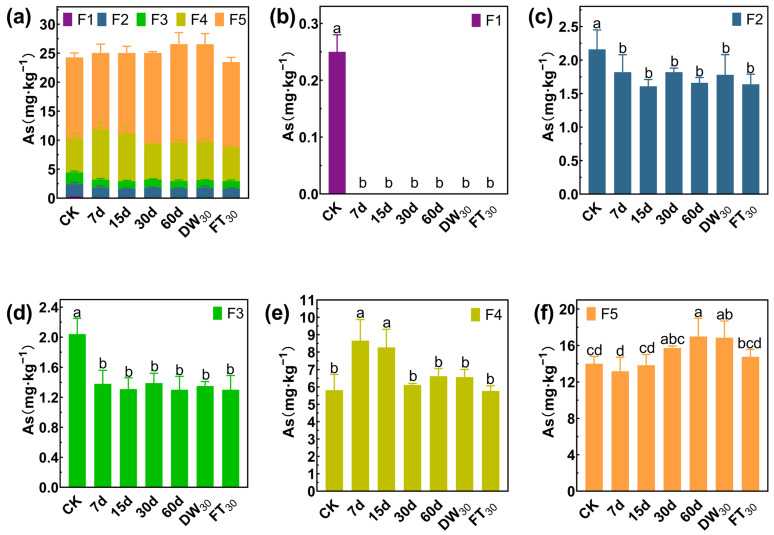
Formation of arsenic in Soil#2: Wenzel extraction. (**a**) Column chart of As speciation distribution in soil; Concentration changes of F1 (**b**), F2 (**c**), F3 (**d**), F4 (**e**) and F5 (**f**) fraction As. CK represents untreated control soil samples without amendment. Samples labeled 7d, 15d, 30d, and 60d denote specimens amended with 5% FMHC and cured for 7, 15, 30, and 60 days, respectively, under natural conditions. DW_30_ and FT_30_ indicate modified soil samples subjected to environmental stress testing through 30 cycles of alternate wet–dry conditions and freeze–thaw treatments, respectively. The five chemical fractions of arsenic are operationally defined as follows: F1: non-specifically sorbed (easily mobilizable, outer-sphere complexes); F2: specifically sorbed (readily mobilizable, inner-sphere complexes); F3: amorphous to poorly crystalline Fe/Al hydrous oxide-bound fraction; F4: well-crystallized Fe/Al hydrous oxide-bound fraction; and F5: residual fraction. Different lowercase letters indicate significant differences between groups (*p* < 0.05).

Arsenic also mainly exists in the forms of F4 and F5, accounting for 65.93% in Soil#1 and 81.67% in Soil#2 (Figure 7 and Figure 8). Arsenic speciation showed more extensive stabilization, with F1 fractions in Soil#1 decreasing from 3.7 mg·kg^−1^ to 0.1–0.2 mg·kg^−1^ (>95% reduction) and in Soil#2 from 0.2 mg·kg^−1^ to <0.01 mg·kg^−1^ (>98% reduction). The F2 fractions were reduced by 21–30% in Soil#1 and 16–25% in Soil#2, while F3 fractions showed modest decreases of <15% and 32–36%, respectively. The treatment effectively converted nearly all F1 arsenic and partial F2/F3 fractions into stable F4/F5 forms, though, as with antimony, the elevated baseline levels of these stable fractions prevented observation of significant concentration increases.

## 4. Discussion

To date, few studies have investigated the potential of pristine and modified hydrochar to stabilize antimony and arsenic in soil. Pristine and iron-modified rice husks were applied to stabilize Sb (66.58 mg·kg^−1^) in farmland soil near the antimony mining area [26]. The iron-modified hydrochar (containing 20.57% Fe) could significantly reduce the bioavailability (exchangeable and carbonate-bound fractions) of Sb by 40%, while the pristine hydrochar immobilized only 7.7%, at the same supplemental dosage (5%). Similarly, iron-encapsulated hydrochar (containing 50% Fe) was used to immobilize As (2580 mg·kg^−1^) in a mining-contaminated soil [27]. At 3% application, As in the mobile phases was reduced by 30% compared to the additive-free control. Like these ion-modified hydrochars, the iron and manganese co-precipitated hydrochar (FMHC) exhibited great performance in alleviating antimony and arsenic in soil.

Taking leaching toxicity as the evaluation index, FMHC can effectively stabilize antimony and arsenic in the soil and shows potential for long-term stability. The stabilization rate remains stable after 30 rounds of accelerated aging treatment, with the stabilization rate for antimony (with initial leaching concentration ranging from 0.3 to 4.5 mg·L^−1^) being higher than 70%. The stabilization rate of arsenic (with an initial leaching concentration less than 0.4 mg·L^−1^) is higher than 94%. Although the leaching concentrations of Sb/As in Soil#1 were an order of magnitude higher than those in Soil#2, FMHC maintained comparable stabilization efficiencies for Sb (70–90%) and As (>94%) in both soils, indicating its performance was largely unaffected by initial contamination levels in the tested soils. In Soil#1, Sb leaching concentrations were an order of magnitude higher than As, while in Soil#2, Sb levels exceeded As by two orders of magnitude, demonstrating incomparable concentration differences between the two metalloids within the same soil. Notably, the As leaching concentration in Soil#1 (156–353 μg·L^−1^) was similar to Sb levels in Soil#2 (288–383 μg·L^−1^), yet FMHC achieved >97% stabilization for As in Soil#1 versus 79–90% for Sb in Soil#2. Specifically, for soils with initial leaching concentrations of 300 μg·L^−1^, post-remediation As levels met drinking water standards (<10 μg·L^−1^), whereas remediated Sb concentrations remained >30 μg·L^−1^ significantly exceeding the WHO guidelines (5 μg·L^−1^). These disparities likely stem from the preferential adsorption of As over Sb by iron oxides [28], revealing FMHC’s limitations in Sb remediation that necessitate combined use with supplementary materials (e.g., aluminum oxides, Ca(OH)_2_) for practical applications [29,30].

When evaluated by leaching toxicity, Fe/Mn-based chemical stabilizers generally achieve effective stabilization of soil Sb and As, with short-term (≤365d) stabilization efficiencies ranging from 28% to 96%, depending on stabilizer dosage and initial leaching toxicity. For instance, application of 0.5–1.5% (*w*/*w*) Fe@H_2_O_2_-BC demonstrated stabilization efficiencies of 90.7–95.7% for Sb (initial H_2_SO_4_-HNO_3_ extractable concentration: 0.05 mg·L^−1^) and 89.6–90.8% for As (0.16 mg·L^−1^) [31]. The application of 1% (*w*/*w*) Fe-Mn-modified biochar to As-contaminated soil reduced TCLP-leachable As concentrations from 3.95 mg·L^−1^ to 0.77 mg·L^−1^, achieving an 80% stabilization efficiency [13]. The application of 5% (*w*/*w*) Fe(II)-activated Fenton sludge simultaneously reduced TCLP-leachable As and Sb concentrations from 12.03 μg·L^−1^ to 2.28 μg·L^−1^ (81% stabilization) and from 292.17 μg·L^−1^ to 91.31 μg·L^−1^ (69% stabilization), respectively [32]. The application of 1% FeMg-modified biochar reduced TCLP-leachable Sb and As concentrations in co-contaminated soils by 28% (from 775 to 554 μg·L^−1^) and 83% (from 166 to 28 μg·L^−1^), respectively [33].

The evaluation of long-term stabilization efficacy for Sb- and As-contaminated soils suffers from inadequate standardized protocols. Although innovative approaches like the “soil coin” method, a quantitative artificial aging technique simulating proton attack on soil amendments, have been developed, these require extensive field validation and calibration [34]. Laboratory-accelerated aging tests (freeze–thaw cycling, wet–dry alternation, acid rain leaching, and biochemical oxidation) remain essential for evaluating long-term stabilization efficacy. However, the effects of freeze–thaw cycling on metalloid leaching exhibit significant context-dependent variations. While freeze–thaw cycling exhibited no significant influence on ZVI-stabilized Sb and As leaching (H_2_SO_4_-HNO_3_ extraction) in alkaline soil (pH 9.1), it significantly reduced the ZVI-stabilized Sb leaching and caused an obvious fluctuation in ZVI-stabilized As leaching in acidic soil (pH 6.0) [35]. Twenty freeze–thaw cycles caused negligible changes in As leaching (synthetic precipitation leaching procedure (SPLP)) across untreated and amended (biochar, Fe-Mn oxides, and biochar-loaded Fe-Mn oxides) soils [36]. Nevertheless, laboratory-accelerated aging remains a valuable preliminary indicator of long-term stabilization performance, albeit with limitations. Furthermore, simple accelerated aging proved that FMHC has the potential to reduce the leaching toxicity of antimony and arsenic in the soil in the long term.

Two standardized extractants are commonly employed to assess the bioavailability of Sb and As in soils: 0.1 M Na_2_HPO_4_ and 0.5 M NaHCO_3_. The application of 2% (*w*/*w*) magnetic graphene-loaded biochar gel to Sb-As co-contaminated soil reduced Na_2_HPO_4_-extractable bioavailable As by 9.9% (initial ~15 mg·kg^−1^) and Sb by 16.4% (initial ~65 mg·kg^−1^) [37], while 1% (*w*/*w*) FeMg-modified biochar reduced Sb by 23.0% (from 15.06 mg·kg^−1^ to 11.6 mg·kg^−1^) and As by 31.1% (from 6.95 mg·kg^−1^ to 4.79 mg·kg^−1^) [33]. The amendment of 0.5–1.5% (*w*/*w*) Fe@H_2_O_2_-BC decreased NaHCO_3_-extractable Sb by 65.0–95.6% (initially ~1.6 mg·kg^−1^) and As by 91.1–96.0% (initially ~7.5 mg·kg^−1^) [31]; meanwhile, 5% (*w*/*w*) Fe(II)-activated Fenton sludge could reduce As (from 7.02 mg·kg^−1^ to 0.8 mg·kg^−1^) by 88.6% and Sb (from 13.65 mg·kg^−1^ to 2.27 mg·kg^−1^) by 83.3% [32]. While Na_2_HPO_4_ and NaHCO_3_ yield comparable Sb extraction efficiencies [23], the stabilization rates calculated using Na_2_HPO_4_ are typically lower than NaHCO_3_-derived values. This divergence arises from the stronger competitive displacement of surface-bound Sb/As by PO_4_^3−^ because of their structural similarities [28]. Furthermore, the above-mentioned differences further prove the limitations of laboratory extraction in evaluating the bioavailability of antimony and arsenic. Future effectiveness experiments should involve plants.

Accelerated aging can alter the physical and chemical properties of soil, thereby affecting the stability of heavy metals via stabilizers. Notably, accelerated aging exhibits dual effects on bioavailable As: The freeze–thaw process can promote the vertical migration of As and increase NaH_2_PO_4_-extractable As in paddy soils [38]. For marine sedimentary As, wet–dry cycles promote “self-induced” fixation, while freeze–thaw cycles increase CaCl_2_-extractable fractions [39]. The influence of accelerated aging on available antimony remains unknown. Therefore, the long-term assessment of bioavailability should be combined with plant growth. Laboratory-accelerated aging cannot provide convincing conclusions.

Generally, higher iron oxide content in soils correlates with lower bioaccessibility of both antimony and arsenic, though other physicochemical properties, including pH, manganese content, and organic matter, also influence their bioaccessibility [40]. For example, natural hematite and goethite have been shown to effectively reduce the bioaccessibility of both As and Sb in calcareous soils [30]. In addition to iron-based stabilizers, biochar and seaweed-derived fertilizers can also decrease antimony bioaccessibility [41]; however, pristine biochar treatment may conversely increase arsenic bioaccessibility in soils [42,43], while zero-valent iron biochar successfully reduces its bioaccessibility [44]. Since the bioaccessibility of soil-borne antimony and arsenic is regulated by multiple competing factors, the application of iron-based materials does not guarantee reduced bioaccessibility in all cases. For instance, while zero-valent iron amendment can reduce arsenic bioaccessibility in both acidic and alkaline soils and decrease antimony bioaccessibility in alkaline soils, it typically shows negligible effects on antimony bioaccessibility in acidic soil environments [35].

**Figure 9:**
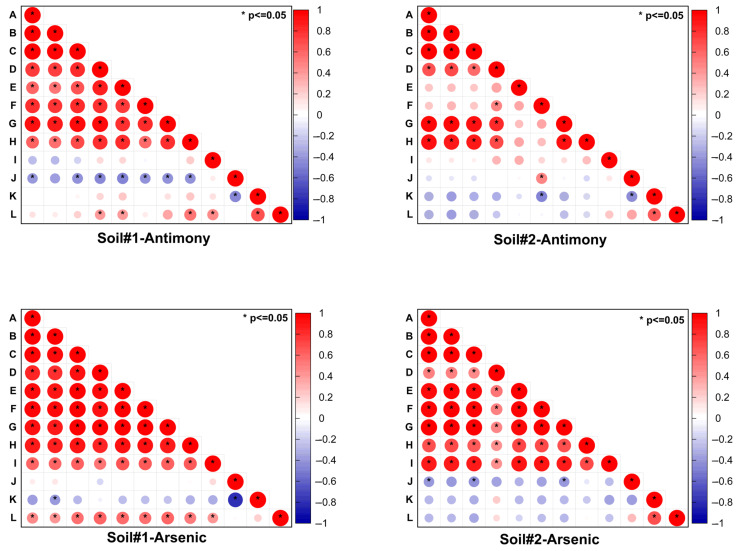
Correlation heatmap of Sb/As concentrations in soils extracted using different methods. Letters A-L represent concentrations of Sb/As extracted via different methods: (A) HNO_3_-H_2_SO_4_ extraction, (B) water extraction, (C) TCLP extraction, (D) bioavailable concentration, (E) PBET-simulated gastric phase extraction, (F) PBET-simulated intestinal phase extraction, (G) F1 fraction (non-specifically sorbed), (H) F2 fraction (specifically sorbed), (I) F3 fraction (amorphous to poorly crystalline Fe/Al hydrous oxide-bound), (J) F4 fraction (well-crystallized Fe/Al hydrous oxide-bound), (K) F5 fraction (residual), and (L) total concentration. Circle size and color intensity both correspond to correlation coefficient magnitude (see color scale). Asterisks (*) denote statistical significance (*p* < 0.05).

A correlation analysis was conducted to compare antimony and arsenic concentrations in both soils obtained via different extraction methods (Figure 9). In Soil#1, significant positive correlations (*p* < 0.05) were observed between leachable/available/bioaccessible antimony concentrations and the F1 and F2 fractions, while arsenic showed similar correlations extending to the F3 fraction. Soil#2 exhibited comparable relationships for both metalloids. A more detailed quantitative analysis revealed that FMHC treatment induced significant changes in Sb and As fractionation in both soils (Table 3). For antimony, we observed marked decreases in the more labile F1 and F2 fractions alongside nearly constant F3 proportions and substantial increases in the stable F4 and F5 fractions. Similarly, arsenic showed reductions in its combined F1-F3 fractions with corresponding increases in the residual F4 and F5 fractions. These transformation patterns were consistently reflected across all five extraction methods (leaching toxicity, bioavailability, and bioaccessibility tests), where Soil#1 consistently demonstrated higher extraction ratios for both Sb and As compared to Soil#2. Importantly, this difference paralleled Soil#1′s initially higher proportions of reactive F1 and F2 fractions, providing compelling evidence that the environmental toxicity of soil antimony and arsenic, as evaluated through in vitro simulated extraction methods, is predominantly controlled by these two most readily and easily mobilizable fractions (F1 and F2). The two investigated soils, though collected from the same abandoned antimony smelting site, exhibited distinct differences in Sb and As speciation patterns, particularly in their readily mobile F1 and F2 fractions. The chemical forms of antimony and arsenic in soils are governed by multiple interacting factors, including (but not limited to) the content and dissolution behavior of iron/manganese/aluminum oxides, dissolved organic matter concentration, clay mineral content, soil pH, and redox potential [45,46,47,48]. Since this study exclusively monitored pH variations during the aging process, we cannot perform further mechanistic analysis regarding the observed differences in Sb/As speciation between the two soils.

These results demonstrate that FMHC could promote metalloid stabilization: for antimony, it primarily converted non-specifically (F1) and specifically adsorbed (F2) forms into stable well-crystallized Fe/Al hydrous oxide-bound (F4) and residual fractions (F5); for arsenic, it also transformed the amorphous to poorly crystalline Fe/Al hydrous oxide-bound fraction (F3) into more stable chemical forms (F4 and F5). The stabilization efficiency varied with soil type and aging conditions, but consistently showed preferential reduction in the most bioavailable fractions (F1–F3) for both metalloids. Iron-based stabilizers generally facilitate the transformation of labile antimony and arsenic into more stable fractions. For instance: magnetic graphene-loaded biochar gel treatment significantly decreased arsenic in the F1 fraction by 18.3% and F3 by 21.8%, while increasing F5-bound arsenic by 10.3%; For antimony, it reduced the F1 fraction by 16.5% and increased F4-bound Sb by 17.9%, with no notable changes in other fractions [37]. Fe(II) activated-Fenton sludge amendment reduced combined F1 + F2 arsenic by 25% and antimony by 45%, while enhancing F5 arsenic from 28.29% to 36.63% and F5 antimony from 28.29% to 32.98% [32]. FeMg-modified biochar application decreased F1 + F2 arsenic by 28.3% and antimony by 45.2%, with F5-bound Sb rising from 84.5% (1294 mg/kg) to 88.4% (1353 mg/kg) and F5 arsenic increasing from 43.3% (38.1 mg/kg) to 57.9% (50.9 mg/kg) [33].

**Figure 10:**
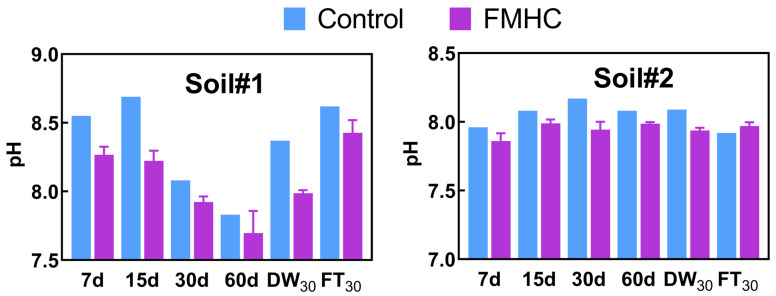
pH of Soil#1 and Soil#2. Control represents untreated control soil samples without amendment, while FMHC represents soils amended with 5% FMHC. 7d, 15d, 30d, and 60d represent soil samples cured for 7, 15, 30, and 60 days, respectively, under natural conditions. DW_30_ and FT_30_ indicate soil samples subjected to environmental stress testing through 30 cycles of alternate wet–dry conditions and freeze–thaw treatments, respectively.

Overall, it is indicated that FMHC could facilitate the transformation of mobile Sb/As fractions into stable states, thus decreasing the leaching toxicity, bioavailability and bioaccessibility of Sb/As in soil. The following potential stabilization mechanisms were tentatively proposed based on the literature. First of all, soil pH value is an important factor affecting the mobility of Sb and As [28]. FMHC amendment persistently acidified soils to varying degrees (ΔpH 0.09–0.47) (Figure 10), which promotes the immobilization of anions As/Sb through electrostatic attraction with the increased positive charge on the surface of soil colloids [37]. Secondly, the protonated carbon and oxygen-containing functional groups (e.g., carboxyl (–COOH) and carbonyl (C=O)) on hydrochar surfaces facilitate Sb/As immobilization through complexation [49,50]. Thirdly, the iron/manganese oxides on the surface of FMHC concurrently stabilize Sb and As through mechanisms including oxidation-reduction, complexation and co-precipitation [6]. The proposed stabilization mechanism is based on preliminary evidence from existing studies. A comprehensive mechanistic analysis would require further experimental validation, including advanced characterization techniques (e.g., X-ray Photoelectron Spectroscopy, Extended X-ray Absorption Fine Structure, or synchrotron-based spectroscopy) to elucidate the specific interactions between the FMHC and Sb/As species.

## 5. Conclusions

This study provides a comprehensive evaluation of Sb and As stabilization performance using iron–manganese-modified hydrochar through integrated assessment of leaching toxicity, bioavailability, and bioaccessibility. The results demonstrate FMHC’s robust stabilization capacity across all three evaluation metrics: (1) leaching toxicity tests revealed excellent immobilization efficiency (>70% for Sb and >94% for As) with remarkable stability during accelerated aging; (2) bioavailability assessment showed maximum reductions of 64% (Sb) and 53% (As), despite some fluctuations; and (3) bioaccessibility analysis demonstrated consistent As stabilization (55–77% reduction) and up to 58% reduction for Sb. Sb immobilization primarily resulted from a decrease in both non-specifically and specifically adsorbed fractions, while As stabilization was accomplished through a reduction in non-specifically/specifically adsorbed and amorphous iron/aluminum oxide-bound fractions. This multi-metric evaluation framework not only confirms FMHC’s effectiveness as a stabilization amendment but also provides valuable insights for developing comprehensive assessment protocols for contaminated soil remediation. The findings offer significant practical implications for managing Sb-As co-contaminated sites, particularly in mining areas, though long-term field validation studies are recommended to verify these promising results under natural conditions to facilitate practical application. While this study successfully demonstrated FMHC’s stabilization performance, three key limitations warrant further investigation: (i) the stabilization mechanisms require more in-depth analysis, (ii) the current single-soil evaluation needs expansion to diverse soil types, and (iii) comprehensive ecotoxicological assessments (e.g., plant uptake tests, soil invertebrate bioassays, and microbial community responses) should be incorporated to validate the ecological safety of this stabilization approach.

## Figures and Tables

**Table 1:** Basic physicochemical properties of Sb/As co-contaminated soil.

Parameters	Soil#1 ^a^	Soil#2 ^a^
pH	8.55	7.96
EC (mS/m)	151.7	150.7
CEC (cmol(+)·kg^−1^)	33.2	32.8
TOC (%)	0.764	0.485
Sand (0.05–2 mm)%	27.4	14
Silt (0.002–0.05 mm)%	52.2	51.2
Clay (<0.002 mm)%	20.3	34.8
Total Sb (mg·kg^−1^)	661.98 ± 71.91	238.69 ± 32.97
Total As (mg·kg^−1^)	51.79 ± 4.21	25.04 ± 2.11

^a^ Soil#1 and Soil#2 were soil samples collected from an antimony smelter located in Gansu, China.

**Table 2:** Extraction methods of antimony and arsenic from soil.

Extraction Agents		Solid/Liquid Ratio	Extraction Conditions
Single-Step Extraction			
HNO_3_-H_2_SO_4_ ^a^	A 2:1 (*v*/*v*) H_2_SO_4_-HNO_3_ mixture was added dropwise to deionized (DI) water to achieve pH 3.20	1.0 g/10 mL	25 °C/30 rpm/18 h
H_2_O ^b^	DI water	1.0 g/10 mL	25 °C/110 rpm/8 h, stand for 16 h
TCLP ^c^	In total, 5.7 mL of glacial acetic acid was diluted to 1 L with distilled water (pH = 2.88)	1.0 g/20 mL	25 °C/30 rpm/18 h
Bioavailability ^d^	0.1 M Na_2_HPO_4_	1.0 g/10 mL	20 °C/200 rpm/2 h
Bioaccessibility PBET ^e^	PBETG (gastric phase): A total of 1.25 g pepsin, 0.5 g sodium malate, and 0.5 g sodium citrate were mixed with 420 μL lactic acid and 500 μL glacial acetic acid. The pH was adjusted to 1.5 with HCl, and the solution was diluted to 1.0 L with DI water	0.4 g/40 mL	37 °C/60 rpm/1 h, stand for 15 min
	PBETI (intestinal phase): A total of 70 mg of bile salts and 20 mg of trypsin were added to 40 mL of the simulated gastric juice extracts, and the pH of the mixture was adjusted to 7.0 with saturated NaHCO_3_		37 °C/60 rpm/4 h
**Wenzel SEP ^f^**			
F1 (Non-specifically sorbed (easily mobilizable, outer-sphere complexes))	0.05 M (NH_4_)_2_SO_4_	1.0 g/25 mL	25 °C/4 h
F2 (Specifically sorbed (readily mobilizable, inner-sphere complexes))	0.05 M NH_4_H_2_PO_4_	1.0 g/25 mL	25 °C/16 h
F3 (Amorphous and poorly crystalline hydrous oxides of Fe and Al)	0.2 M NH_4_-oxalate (pH = 3.25)	1.0 g/25 mL	25 °C/4 h
F4 (Well-crystallized hydrous oxides of Fe and Al)	0.2 M NH_4_-oxalate + 0.1 M ascorbic (pH = 3.25)	1.0 g/25 mL	96 °C/0.5 h
F5 (Residual)	HF–HNO_3_–HClO_4_ (*v*/*v*/*v* = 1:3:2)	0.1 g/50 mL	Digestion under 180 °C, near dryness

^a^ Environmental protection industry standards of the People’s Republic of China, HJ/T 299-2007, solid waste-extraction procedure for leaching toxicity: sulfuric acid and nitric acid method [20]. ^b^ Environmental protection standards of the People’s Republic of China, HJ 557-2010, solid waste-extraction procedure for leaching toxicity: horizontal vibration method [21]. ^c^ U.S. Environmental Protection Agency, Hazardous Waste Test Methods/SW-846 Method 1311: toxicity characteristic leaching procedure [22]. ^d^ The methodology for determining Sb/As bioavailability in soil was adapted from the experimental protocol established by [23]. ^e^ The oral bioaccessibility of Sb and As in contaminated soil was quantitatively assessed using the Physiologically Based Extraction Test (PBET), an in vitro methodology simulating human gastrointestinal conditions to estimate heavy metal(loid) exposure risks [24]. ^f^ Fractionation of Sb/As in soil was analyzed with an improved sequential extraction procedure [25].

**Table 3:** Extraction ratios of antimony and arsenic from soil before and after FMHC amendment.

Proportion of Extractable Part in Total Amount								
Extraction	Soil#1-Sb		Soil#2-Sb		Soil#1-As		Soil#2-As	
	Control ^a^	FMHC ^b^	Control ^a^	FMHC ^b^	Control ^a^	FMHC ^b^	Control ^a^	FMHC ^b^
H_2_O	6.77%	1.27%	1.52%	0.22%	6.55%	0.22%	0.28%	0.01%
HNO_3_-H_2_SO_4_	4.99%	1.09%	1.29%	0.20%	4.89%	0.20%	0.20%	0.01%
TCLP	8.35%	1.84%	2.71%	0.49%	5.72%	0.49%	1.38%	0.03%
Na_2_HPO_4_	6.51%	3.59%	3.05%	1.92%	15.07%	1.92%	6.11%	4.95%
PBET-G	20.04%	12.72%	9.62%	7.37%	14.65%	7.37%	9.30%	3.70%
PBET-I	20.88%	12.44%	10.31%	8.38%	13.99%	8.38%	8.46%	2.07%
F1	3.36%	1.16%	1.28%	0.45%	6.70%	0.45%	1.03%	0.005%
F2	9.92%	5.69%	3.33%	1.74%	18.52%	1.74%	8.88%	6.82%
F3	8.10%	9.12%	5.83%	5.24%	9.01%	5.24%	8.41%	5.30%
F4	34.74%	39.91%	54.05%	51.81%	30.76%	51.81%	23.89%	27.74%
F5	35.51%	40.76%	35.51%	40.76%	57.79%	40.76%	57.79%	60.13%

^a^ Control refers to the untreated soil, and the data was the average of 6 repeats. ^b^ FMHC refers to soil amended with 5% FMHC, and the data was the average of 18 repeats, from 6 treatment groups, with 3 repeats per group.

## Data Availability

Data will be made available on request.
